# Cartography of Pathway Signal Perturbations Identifies Distinct Molecular Pathomechanisms in Malignant and Chronic Lung Diseases

**DOI:** 10.3389/fgene.2016.00079

**Published:** 2016-05-06

**Authors:** Arsen Arakelyan, Lilit Nersisyan, Martin Petrek, Henry Löffler-Wirth, Hans Binder

**Affiliations:** ^1^Group of Bioinformatics, Institute of Molecular Biology, National Academy of SciencesYerevan, Armenia; ^2^College of Science and Engineering, American University of ArmeniaYerevan, Armenia; ^3^Laboratory of Immunogenomics, Department of Pathological Physiology, Faculty of Medicine and Dentistry, Institute of Molecular and Translational Medicine, Palacky University OlomoucOlomouc, Czech Republic; ^4^Interdisciplinary Centre for Bioinformatics, University of LeipzigLeipzig, Germany

**Keywords:** high-throughput gene expression, biological pathways, pathway signal flow, self-organizing maps, chronic lung diseases, lung cancers

## Abstract

Lung diseases are described by a wide variety of developmental mechanisms and clinical manifestations. Accurate classification and diagnosis of lung diseases are the bases for development of effective treatments. While extensive studies are conducted toward characterization of various lung diseases at molecular level, no systematic approach has been developed so far. Here we have applied a methodology for pathway-centered mining of high throughput gene expression data to describe a wide range of lung diseases in the light of shared and specific pathway activity profiles. We have applied an algorithm combining a Pathway Signal Flow (PSF) algorithm for estimation of pathway activity deregulation states in lung diseases and malignancies, and a Self Organizing Maps algorithm for classification and clustering of the pathway activity profiles. The analysis results allowed clearly distinguish between cancer and non-cancer lung diseases. Lung cancers were characterized by pathways implicated in cell proliferation, metabolism, while non-malignant lung diseases were characterized by deregulations in pathways involved in immune/inflammatory response and fibrotic tissue remodeling. In contrast to lung malignancies, chronic lung diseases had relatively heterogeneous pathway deregulation profiles. We identified three groups of interstitial lung diseases and showed that the development of characteristic pathological processes, such as fibrosis, can be initiated by deregulations in different signaling pathways. In conclusion, this paper describes the pathobiology of lung diseases from systems viewpoint using pathway centered high-dimensional data mining approach. Our results contribute largely to current understanding of pathological events in lung cancers and non-malignant lung diseases. Moreover, this paper provides new insight into molecular mechanisms of a number of interstitial lung diseases that have been studied to a lesser extent.

## Introduction

High-throughput gene expression profiling has found wide applications in many areas of lung pathology research, diagnostics, and treatment (Campbell et al., 2011). It has been used for development of biomarker panels allowing for accurate discrimination between diseases, such as chronic obstructive pulmonary disease (COPD), idiopathic pulmonary fibrosis (IPF), and lung cancers (Selman et al., 2006; Wang et al., 2008). The study of gene expression signatures largely contributed to better understanding of molecular pathology of lung diseases(Cancer and Atlas, 2012; Thakur et al., 2014), and to identification of new disease subclasses/entities (Bhattacharjee et al., 2001; West et al., 2012; Li et al., 2014). It also provided new approaches to diagnostics (Buettner et al., 2013; DePianto et al., 2015), and helped to suggest novel therapeutic compounds (Campbell et al., 2012; Gerber et al., 2015). However, most of these studies have been performed in a gene centered fashion, where biological function mining was limited to identification of differentially expressed genes and to enrichment analysis of the obtained gene lists in Gene Ontology (GO), Kyoto Encyclopedia of Genes and Genomes (KEGG) or any other functional annotation database (Arakelyan et al., 2013). Such simple functional annotations of differentially expressed genes might however have overlooked important information, such as interactions between the components in a biological system and their functional consequences. In this regard, explicit assessment of activity regulation in biological pathways by combining expression data of genes with knowledge about the interactions between their products would represent a more straightforward approach. Biological pathways are sequences of physical molecular interactions that guide information propagations (also called signal flow) leading to regulatory consequences for cell function. Pathways are often branched and have more than one target processes, meaning that activation of different sets of genes within the same pathway may lead to different outcomes. Moreover, genes can be involved in more than one pathway, because different pathways may share common branches, sources, and sinks (Daigle et al., 2010). It has been recognized that pathogenesis of diseases usually involve perturbations occurring at pathways level (Logan and Nusse, 2004; Courtois and Gilmore, 2006). Thus, the explicit analysis of pathway activity deregulation is expected to lead to better understanding of molecular pathomechanisms of diseases.

Pathologic characteristics of lung diseases are very complex due to the involvement of environmental and genetic interactions (Pouladi et al., 2015) and do not always reflect the underlying molecular mechanisms. Dysfunction of a single gene may contribute to multiple lung diseases leading to development of different phenotypes or, vice versa, similar disease phenotypes can be caused by dysfunctions of different genes and (Lewis et al., 2008; Pennings et al., 2008). For example, the WNT signaling pathway has been shown to be oppositely involved in the pathobiology of COPD and IPF. Despite this difference, both diseases end up with alveolar senescence and lung “premature aging” (Chilosi et al., 2012). Contrarily, typical interstitial lung diseases, such as sarcoidosis and IPF, largely share similarly deregulated genes, especially in extracellular signaling pathways, meanwhile demonstrating significant differences in downstream signaling pathways (Leng et al., 2013).

In this study we have applied a pathway-centered method for mining high-throughput gene expression data aimed at extracting knowledge about pathobiology of a wide spectrum of malignant and chronic lung diseases by providing an extended systems view on pathway deregulation states. We, used a previously developed and intensively benchmarked data mining approach for gene expression analysis using self-organizing maps machine learning (Wirth et al., 2011) in combination with the pathway signal flow (PSF) algorithm, which maps gene expressions to pathway topologies to assess the strength of the signals that propagate along the pathways (Arakelyan et al., 2013; Binder et al., 2014; Nersisyan et al., 2015). It allowed for combining lung diseases into groups of common pathway perturbations and provided new insights on shared and specific pathomechanisms of lung diseases.

## Materials and methods

### Data and sources

**Figure 1:**
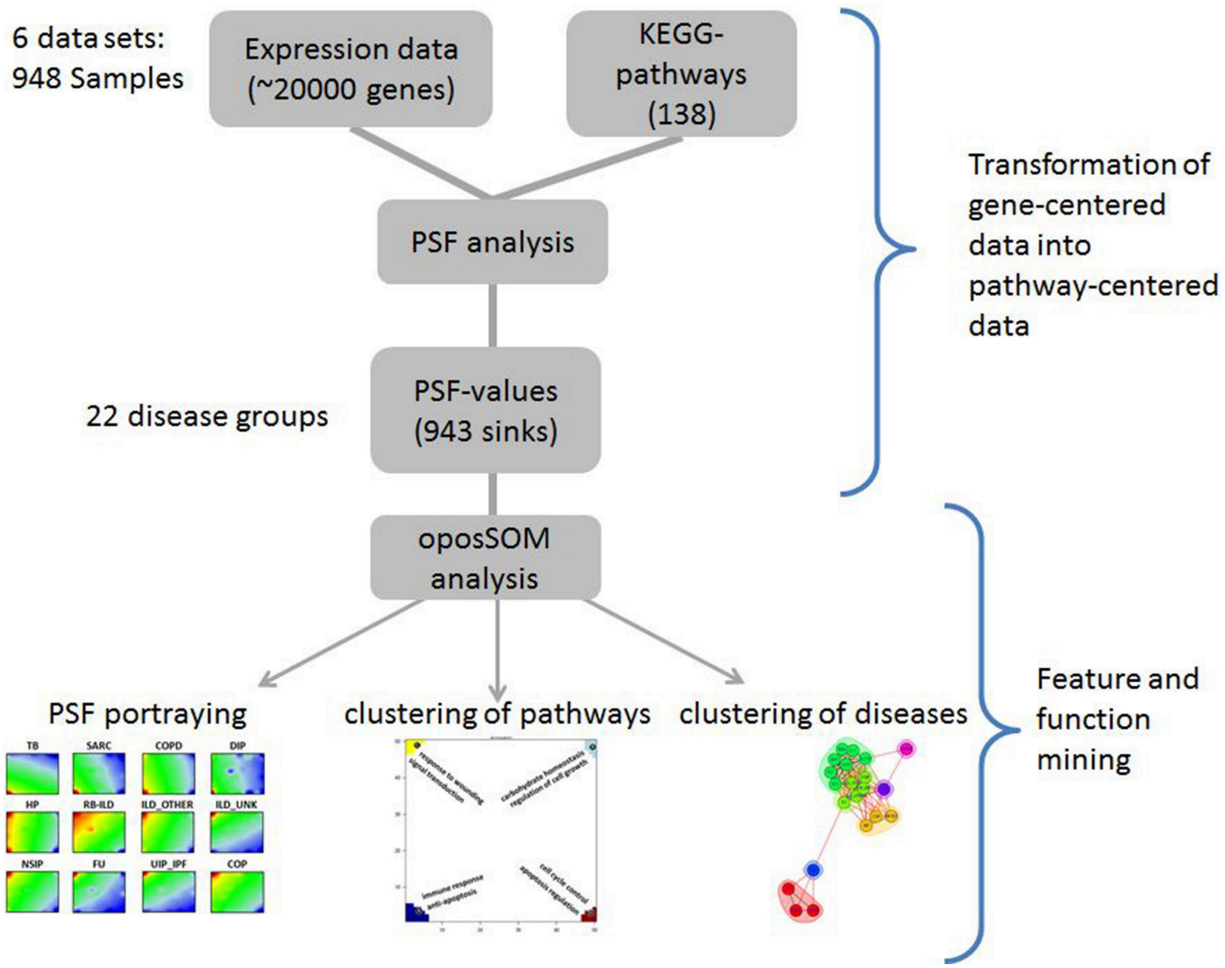
**Workflow of the PSF-SOM method as applied to lung diseases**. Expression data were analyzed in terms of pathway signal flows (PSF) in a series of KEGG canonical pathways. The PSF values of selected sink nodes in the pathways were then clustered using SOM machine learning. This method provides individual “portraits” of sink-node activities of each disease class. Similarities between them were studied using different clustering methods. For details please refer to the Methods section of the manuscript.

Six datasets on selected lung diseases extracted from the Gene Expression Omnibus (GEO) public repository were used (Edgar et al., 2002; Barrett et al., 2011). The datasets were downloaded from GEO in the form of Series Matrix Files, which contain metadata about samples, array calibration and normalization methods and actual normalized data, stored in the form of gene expression matrices, where probe IDs are rows and samples are columns (see Supplementary Material Data Sheet 1, Table S1). From these datasets only samples obtained from untreated patients were used in downstream analyses. In total, 948 diseased and normal lung samples constituting 21 disease groups and one healthy lung tissue were included in the analyses (Table 1). The analysis workflow is presented in Figure 1. It was performed using the R-program package oposSOM (Löffler-Wirth et al., 2015) and a series of stand-alone R-scripts (Supplementary Material Data Sheet 2).

**Table 1:** **Lung diseases and sample sizes used in this study**.

***N***	**Class**	**Lung disease and control group**	**Abbreviation**	**Number of samples**
1	Healthy control	Healthy Control	HC	170
2	Chronic lung diseases	Tuberculosis	TB	5
3		Acute interstitial pneumonia	AIP	1
4		Cryptogenic organizing pneumonia	COP	3
5		Chronic obstructive pulmonary disease	COPD	220
6		Desquamative interstitial pneumonia	DIP	4
7		Fibrosis unknown	FU	10
8		Hypersensitive pneumonitis	HP	30
9		Interstitial lung disease other	ILD_OTHER	9
10		Interstitial lung disease unknown	ILD_UNK	14
11		Nonspecific interstitial pneumonia	NSIP	14
12		Respiratory bronchiolitis-interstitial lung disease	RB-ILD	12
13		Pulmonary sarcoidosis	SARC	6
14		Idiopathic pulmonary fibrosis	UIP_IPF	157
15	Lung cancers	Lung adenocarcinoma	ADC	85
16		Lung cancer basaloid	BAS	39
17		Lung cancer carcinoid	CARCI	24
18		Large cell carcinoma	LCC	3
19		Lung cancer large cell neuroendocrine	LCNE	56
20		Lung cancer other	LCO	4
21		Small cell lung carcinoma	SCC	21
22		Squamous cell carcinoma	SQC	61

### Data preprocessing

For each dataset, microarray probe IDs were converted into Entrez Gene IDs. Microarray probes that did not match any known Entrez ID were discarded. Multiple probe sets for the same gene were averaged. Next, gene expression values in each dataset were inspected for data transformation type (log2, log10 transformation) using a modified version of the “GEO2R log autocheck” script (https://www.ncbi.nlm.nih.gov/geo/geo2r/) and were brought to a linear scale. Then we have converted all values to fold change (FC) respective to the mean expression value of the controls (healthy samples) included in the same dataset. These steps cast the data in all datasets to a common scale and type.

### Pathway signal flow calculation

Assessment of pathway activity deregulation was performed using the PSF algorithm described in detail elsewhere (Arakelyan et al., 2013; Binder et al., 2014; Nersisyan et al., 2015). Briefly, this algorithm computes the strength of the signal propagated from the pathway input to the output through interactions of pathway component genes, based on their fold change expression values. Human reference pathway maps were retrieved from the KEGG Pathway database (Kanehisa, 2002). From 291 KEGG Pathways, 138 KEGG metabolic, signaling and organismal pathway maps were selected by exclusion of disease and drug response specific pathways, as well as pathways lacking annotated interactions (for example ko03010, Ribosome). FC values for each gene were mapped to corresponding pathway nodes, and were averaged if a node contained more than one gene. After this step an input signal of unity was applied to the pathway source nodes. Then PSF values were calculated at the output nodes. PSF algorithm is calibrated in a way that gene expression of FC = 1 at all nodes (normal gene expression) produces PSF = 1 values. Values of PSF less than unity refer to pathway de-activation, while PSF > 1 indicates pathway activation.

**Figure 2:**
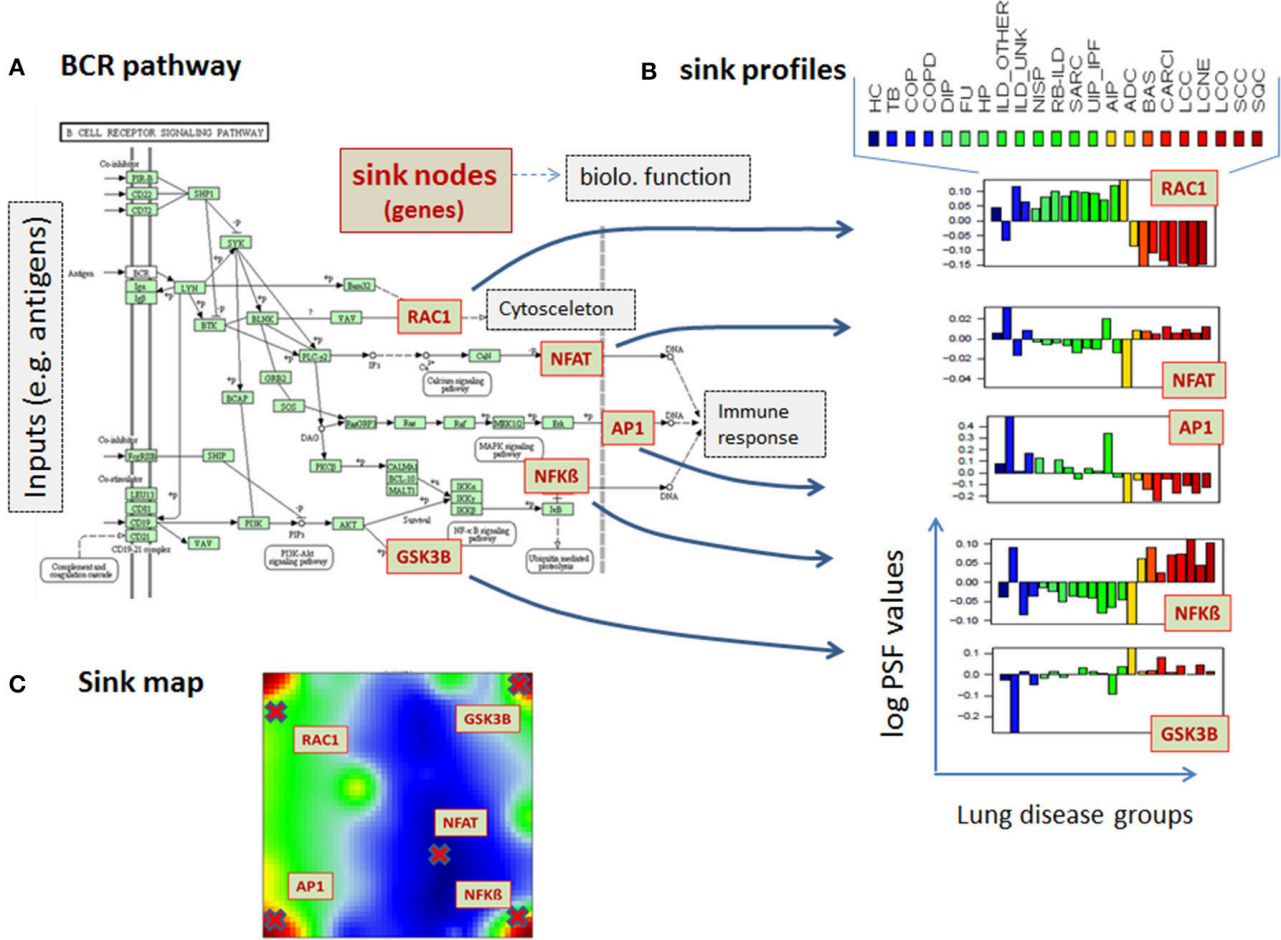
**PSF-profiles of the KEGG B cell signaling (BCR) pathway. (A)** Red nodes in the BCR pathway represent pathway sinks. Their PSF values are shown in part **(B)** for all disease classes studied (see Table 1 for assignments). PSF values depend on the gene expression of the genes upstream from the sink nodes (green boxes in the pathway) which change in a disease specific fashion. **(C)** The location of the sink node genes in the summary SOM map.

Pathways are usually highly branched and can have multiple inputs and outputs, called sources and sinks, respectively. The sinks can be associated with defined biological function and they usually weakly depend on the activity of other sinks in the same pathway. For example, the B cell receptor (BCR) signaling pathway gets input signals from ligands and provides output signals via the sinks ***Rac***, which is associated with *Regulation of actin cytoskeleton pathway*, ***NFAT***, ***AP1***, and ***NFkB***, which lead to expression of genes involved in immune response, and ***GSN3B***, which is a protein phosphorylating enzyme (Figure 2A). Different disease-specific expression values of genes involved in this pathway may lead to different PSF values at the sink nodes and thus, also to different activities of the associated molecular processes. For example, the Rac-sink of the B cell receptor pathway ends up with low activities in lung cancers (red bars Figure 2B) and high activities in chronic lung diseases (green); whereas the NFkB-sink shows nearly an anticorrelated activity profile compared to that of Rac (Figure 2B). In total, for each sample included in the analyses, we calculated PSF values for 943 sinks in 138 pathways (on average, seven sinks per pathway).

### Pathway signal flow—self organizing maps analysis (PSF-SOM)

The self-organizing maps (SOM) method was developed in the early 1980'ties by T. Kohonen (Kohonen, 1982). First applications to microarray gene expression data were published in 1999 (Tamayo et al., 1999; Törönen et al., 1999), to cluster gene expression profiles into a predefined small number of groups of similarly expressed genes. A sample-centered clustering approach was realized shortly after providing a visual identity of the expression landscapes of each sample (Golub et al., 1999; Covell et al., 2003; Eichler et al., 2003). Several methodical improvements of the SOM-technique were developed enabling more flexible learning and mapping in different applications (see Binder and Wirth, 2014 and references cited therein). We pursued a special implementation of SOM that we called SOM portraying (Wirth et al., 2011; Hopp et al., 2013). It visualizes the “landscapes” of large scale -omics data in a comprehensive and intuitive fashion. This method combines both the sample- and gene-centered perspectives to decode molecular patterns within a two-dimensional image (Huang et al., 2005). It emphasizes easy sample-to-sample comparison and the identification of important characteristics by direct visual inspection. We amended this method by a sophisticated analysis workflow for downstream analyses including differential feature selection, diversity analysis, function mining, and class discovery in high-dimensional molecular-biological data (Wirth et al., 2011; Hopp et al., 2013). In a series of case studies we applied this method to disentangle cancer subtypes (Hopp et al., 2013), for combined mRNA and miRNA expression analysis (Wirth et al., 2014), and to analyze proteome (Wirth et al., 2012b; Binder et al., 2014), genomic (Binder and Wirth, 2014), DNA-methylation (Hopp et al., 2015), and epigenome (Steiner et al., 2012) landscapes in the context of cancer, healthy populations, microorganisms, tissue, and cell experiments. Strengths of SOM-portraying applications to molecular omics data are dimensionality-reduction and intuitive visualization of high-dimensional data, “personalized” views and detection of outliers and methodical biases in the data and their classification owing to re-weighting in SOM-space (Binder et al., 2015). Disadvantages often ascribed to SOM machine learning such as high computational efforts, loss of resolution, and the problematic choice and adjustment of parameters defining SOM space (e.g., size, topology, boundary, and initiating conditions) and the non-linear data scaling are of minor importance in our applications to molecular *-omics* data. In oposSOM package typical SOM runs require from minutes to, at maximum a few hours of computer time. SOM-space was optimized in extensive pre-studies (see Binder and Wirth, 2014 and references cited therein), performed in a similar and thus comparable way in a large number of applications. Here we combined the SOM portraying method with PSF analysis to achieve a pathway-centered view on pathomechanisms of lung diseases.PSF analysis generated profiles of PSF values for all 943 sinks of all the pathways across all the samples studied. The PSF values of each sink were centralized with respect to the sink-wise global mean over all samples to focus rather on changes of PSF-values than on absolute PSF levels.

We used SOM machine learning implemented in “som” R package as a core for oposSOM package (Löffler-Wirth et al., 2015) to disentangle the multivariate structure inherent in the PSF-data (Wirth et al., 2011). The SOM algorithm arranged PSF profiles onto two-dimensional 50 × 50 grids (maps), where similar profiles are combined into micro-clusters called meta-PSFs, by analogy with meta-genes in the original application of the algorithm to gene expression data. The similarity of PSF-profiles and SOM node weights was calculated using the Euclidean distance formula. The SOM learning rate was set to 0.02, the constant in the inverse learning rate function was set to 0.01 and the radius of the neighborhood was 3. SOM was trained using default parameters implemented in “som” package which is comprised of 12 training epochs for a complete dataset and required about 9 h on a personal computer (core-i5, 8 Gb RAM). This SOM configuration enables the robust identification of spot modules inherent in the data (Wirth et al., 2011).

PSF landscape of each disease group is described by the meta-PSF expression values (“personal” SOM portrait). They are arranged according to the underlying SOM grid and visualized by an appropriate color gradient. The color patterns emerge as smooth textures representing the fingerprint of pathway activity perturbation for each disease. The individual portraits are mutually comparable. The SOM algorithm arranges similar meta-PSF profiles in neighboring tiles of the map whereas more different ones are located more distantly. Meta-PSFs located in the same spot-like region of the map are concertedly deregulated across the diseases. Overexpression (underexpression) spots in each “personal SOM” portrait were determined as clusters of meta-PSFs, which have values above (below) the predefined threshold (90 percent of meta-PSF extremes), or by applying K-means or correlation clustering to the meta-PSF profiles as described in detail elsewhere (Wirth et al., 2011). All spots detected in the individual portraits are transferred into one master map to visualize the global spot patterns.

Significance analysis for differential PSF-values use a shrinkage *t*-test and false discovery rate (FDR) estimation for multiple test correction as implemented in oposSOM and described in detail elsewhere (Wirth et al., 2011, 2012a). This analysis provided lists of pathway sinks with co-regulated activities in the lung diseases and healthy controls studied.

A separate part of analyses focused on assessment of pathway activity changes in lung diseases compared to healthy lungs. For this, the difference of mean-centered PSF values between diseases and healthy controls was computed (PSF_*disease*_–PSF_*healthy lung*_), representing log fold difference of pathway deregulation in diseased over healthy state. A pathway was considered as significantly deregulated, if its p value was less than 0.05 and FDR qvalue was less than 0.2.

### Clustering of diseases based on the pathway activation states

Similarity analysis of lung diseases was performed based on their SOM portraits using so-called second level SOM, independent component analysis (ICA), and hierarchical clustering methods implemented in oposSOM (Wirth et al., 2011). In addition we applied graph community search with random walktrap algorithm implemented in igraph R package using Fruchterman–Reingold layout (Csardi and Nepusz, 2006).

## Results

### Pathway activity portraits of lung diseases

**Figure 3:**
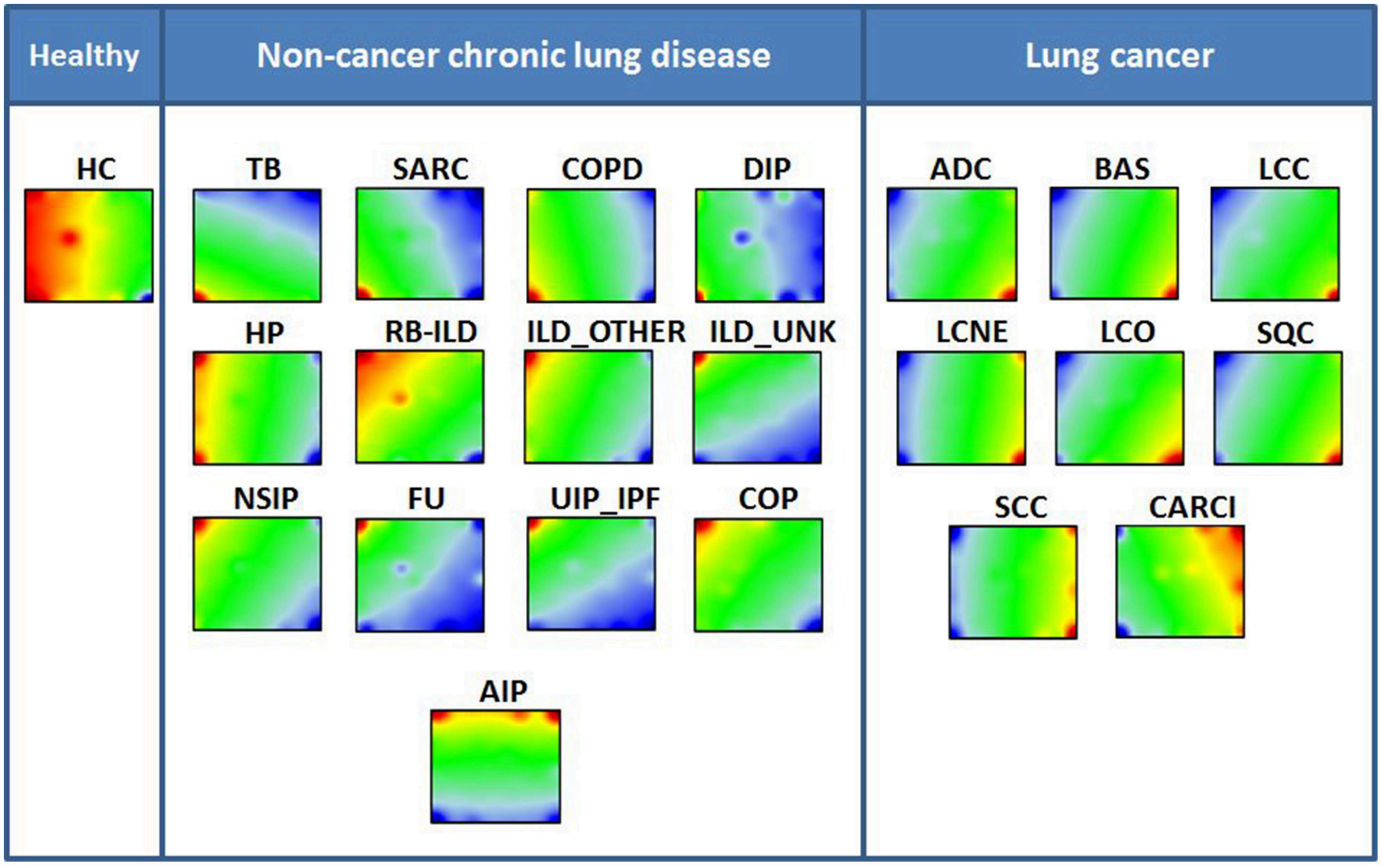
**Disease specific SOM portraits**. Each lung disease and healthy control is characterized by its PSF portrait visualizing the pathway activities. The diseases are sorted into three groups. The color key for the summary SOM portrait is blue-yellow-red, where blue colors represent lowest values, green-yellow transition includes intermediate values and the red colors represent highest values. Note the marked difference between groups, especially between cancer and chronic lung diseases.

PSF profiles were evaluated for 138 KEGG metabolic, signaling, and organismal pathways in 948 diseased and normal lung samples constituting 21 disease groups and one healthy lung tissue (Table 1). Each of the samples was characterized by its state of pathway (de-regulation), which is defined as a set of PSF values at all 943 pathway sinks. In order to evaluate similarities and differences between the states of pathway deregulation of diseased and healthy lungs, we applied machine learning using self-organizing maps (SOM). This method transforms the multidimensional PSF data into a series of two-dimensional images, called “portraits,” which visualize the activities of the sink nodes in each disease studied (Figure 3). These PSF-activity portraits show blue and red spot like regions corresponding to down- and up-regulated sink nodes, respectively. The complete list of pathway sinks associated with each spot in each disease groups in presented in Supplementary Material Data Sheet 3.

It is apparent that healthy lung and non-cancer diseases have clearly distinct pathway deregulation patterns compared to lung cancers (Figure 3). Most cancer diseases are characterized by an upregulated red spot located in the right lower corner, in combination with a down regulated blue spot in the left upper corner of the portrait. Non-malignant lung diseases, in turn, show a distribution of up- and down-regulated spots that virtually mirror that of cancer diseases. Further, this group of diseases showed higher variability of their spot distributions compared to cancers. Here, generally, two different patterns in portraits can be distinguished. One pattern can be specified by the presence of an upregulated spot in the bottom left corner (TB, SARC, COPD, DIP), while the second pattern is defined by an upregulated spot in the upper left corner (NSIP, FU, UIP/IPF, COP, ILD_OTHER, ILD_UNK, RB_ILD). Meanwhile, the spot distribution in HP portrait shows transition between the first and the second chronic lung disease patterns, while the spot distribution on the AIP portrait can be considered as a transition between non-cancer and cancer disease patterns. Finally, it is worthy to note that FU, UIP/IPF, and ILD_UNK share a down-regulated spot with lung malignancies at the bottom left corner of the portraits.

**Figure 4:**
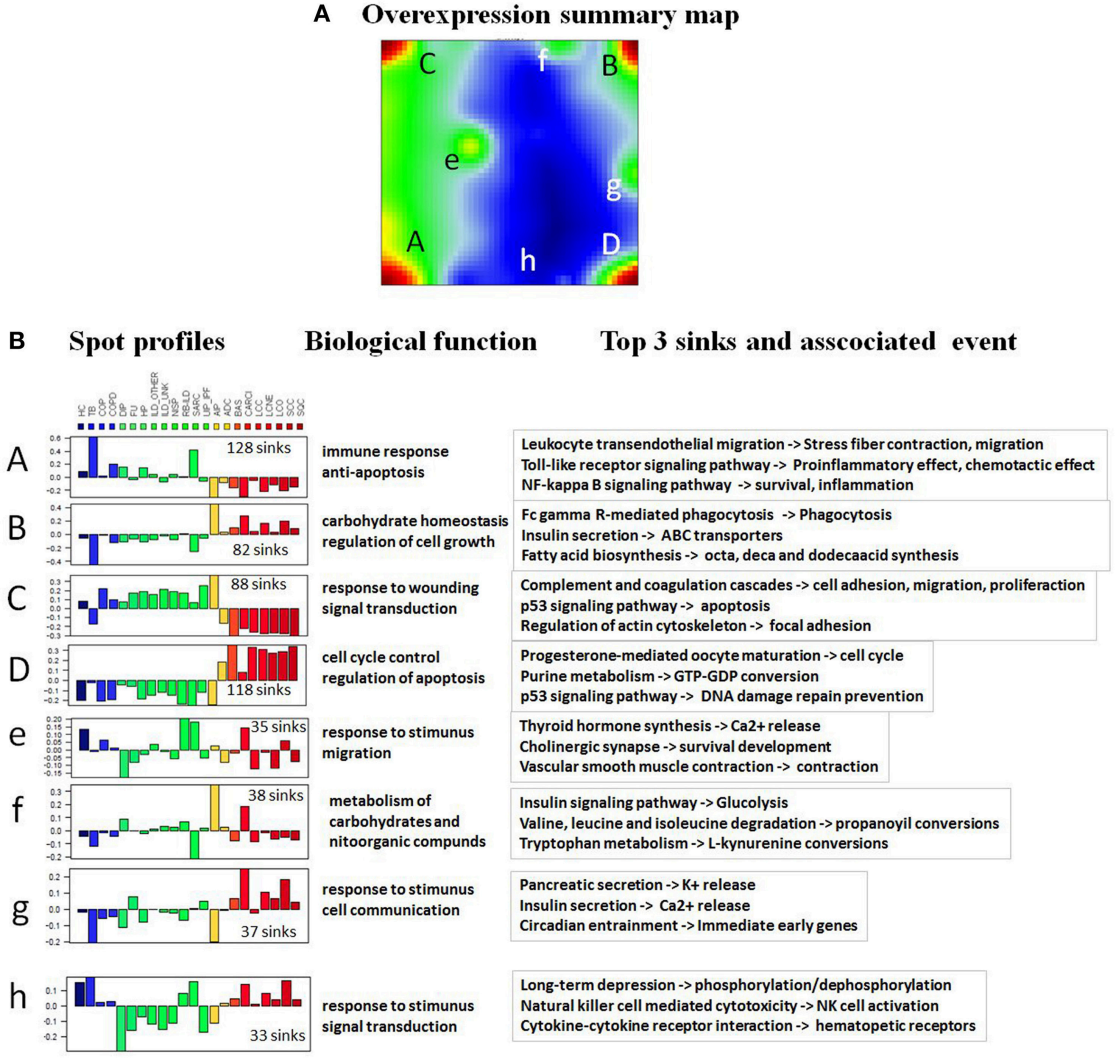
**SOM characteristics. (A)** The overexpression spot summary map reveals four main “spot-like” regions of high PSF activities of the sink nodes in red (A–D), three on intermediate level in green (e–g) and one node with low activity in blue (h). **(B)** Each spot-cluster is characterized by its PSF-profile subsuming about hundred individual sinks. The color key for the summary SOM portrait is blue-yellow-red, where blue colors represent lowest values, green-yellow transition includes intermediate values and the red colors represent highest values. Their main biological function and the top three sink nodes are given in the right part of the figure.

To have a detailed look on pathways involved in formation of these disease-specific portraits, we referred to the overexpression summary map in Figure 4A. It integrates spots from all individual lung disease portraits and thus it provides an overview of all relevant regions becoming activated in the data set. The spots represent clusters of correlated and thus concertedly deregulated pathway sink profiles in one or several diseases. The overexpression spots (A, B, C, and D) on the corners of the summary map represent the main features allowing for distinguishing between non-cancer and malignant lung diseases. The rest of the spots provide finer structure of pathway activation states in different diseases. Since the amplitude of meta-PSF values of these intermediate spots was much lower than that of the major overexpression spots, we decided to concentrate on the latter's in subsequent analyses. Overall, 51 pathways characterized with at least one deregulated sink have been found to be associated with these main overexpression spots (Supplementary Material Data Sheet 1, Table S2). Next, we proceeded to more thorough functional annotation of all pathway sinks located in each overexpression spot of the summary map. Because pathway sinks are gene products directly associated with some functional event or molecular process, we have performed GO term enrichment analysis of sink genes in spots using WebGestalt (Zhang et al., 2005; Figure 4B). The results showed that pathway sinks associated with spots A and C (upregulated in non-cancer diseases) are mainly enriched with GO terms related to immune/inflammatory response, proliferation, and anti-apoptosis, while spots B and D (upregulated in cancer diseases) are linked to cell cycle regulation, apoptosis, and carbohydrate metabolism. The complete list of GO terms associated with pathway sinks and the enrichment significance can be found in the Supplementary Material Data Sheets 4–11.

**Figure 5:**
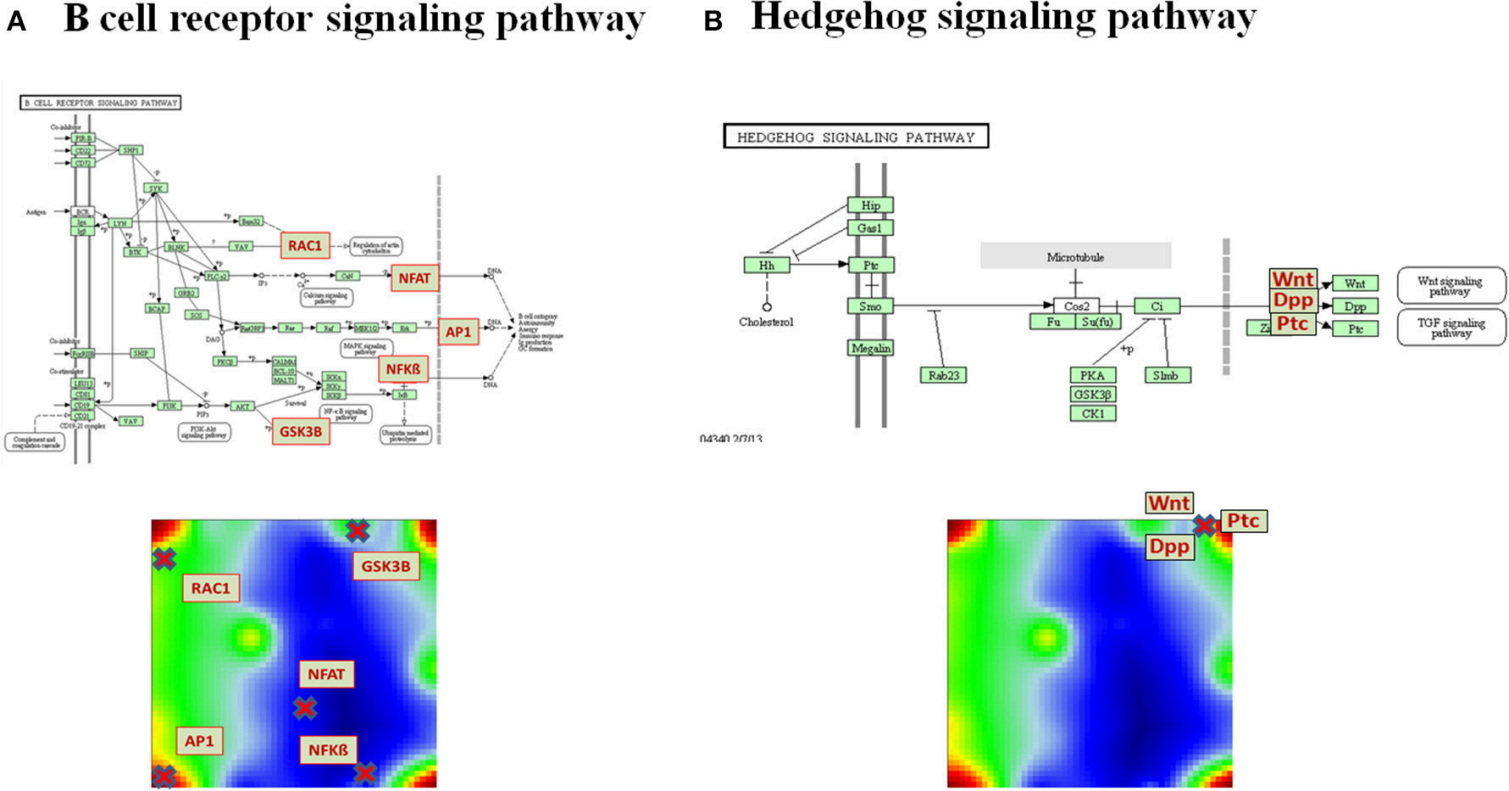
**Distribution of pathway sinks on the SOM map depending on pathway topology. (A)** The sink nodes NFBkB and RAC1 of the BCR pathway are located in opposite corners of the portrait reflecting their anticorrelated profiles. **(B)** In contrast, the sinks of the unbranched Hedgehog signaling pathway are located in the same position on the SOM portrait.

The pathways “Cytokine-cytokine receptor interaction,” “MAPK signaling pathway,” “Cell adhesion molecules (CAMs),” “FoxO signaling pathway” and “Dopaminergic synapse” were found to be significantly associated with all the overexpression spots and thus all the investigated lung conditions; however, with disease-specific molecular processes associated with each sink in these pathways (for details on these pathways and their specific sinks, associated with each spot and their significance values, see to Supplementary Material Data Sheet 1, Table S3). While pathway sinks associated with immune response were located in spots A and C and activated mainly in non-malignant diseases, the cancer-associated spots B and D contained pathway sinks related to signaling, proliferation, and cancer related cell communication. These results show that different sink nodes of a pathway can be located in the same spot or in different ones reflecting thus correlated, anti-correlated, or non-correlated profiles. The examples in Figure 5 illustrate two different situations. Difference of sink profiles in the same pathway can be caused by multiple inputs, multibranching, existence of inhibitory interactions, and/or deregulated expression of intermediate nodes (Figure 5A). On the other hand, unbranched pathway topologies tend to show more concerted profiles of their sink nodes, which, in consequence, accumulate in the same region of the map (Figure 5B).

### Clustering of disease groups based on pathway deregulation activities

**Figure 6:**
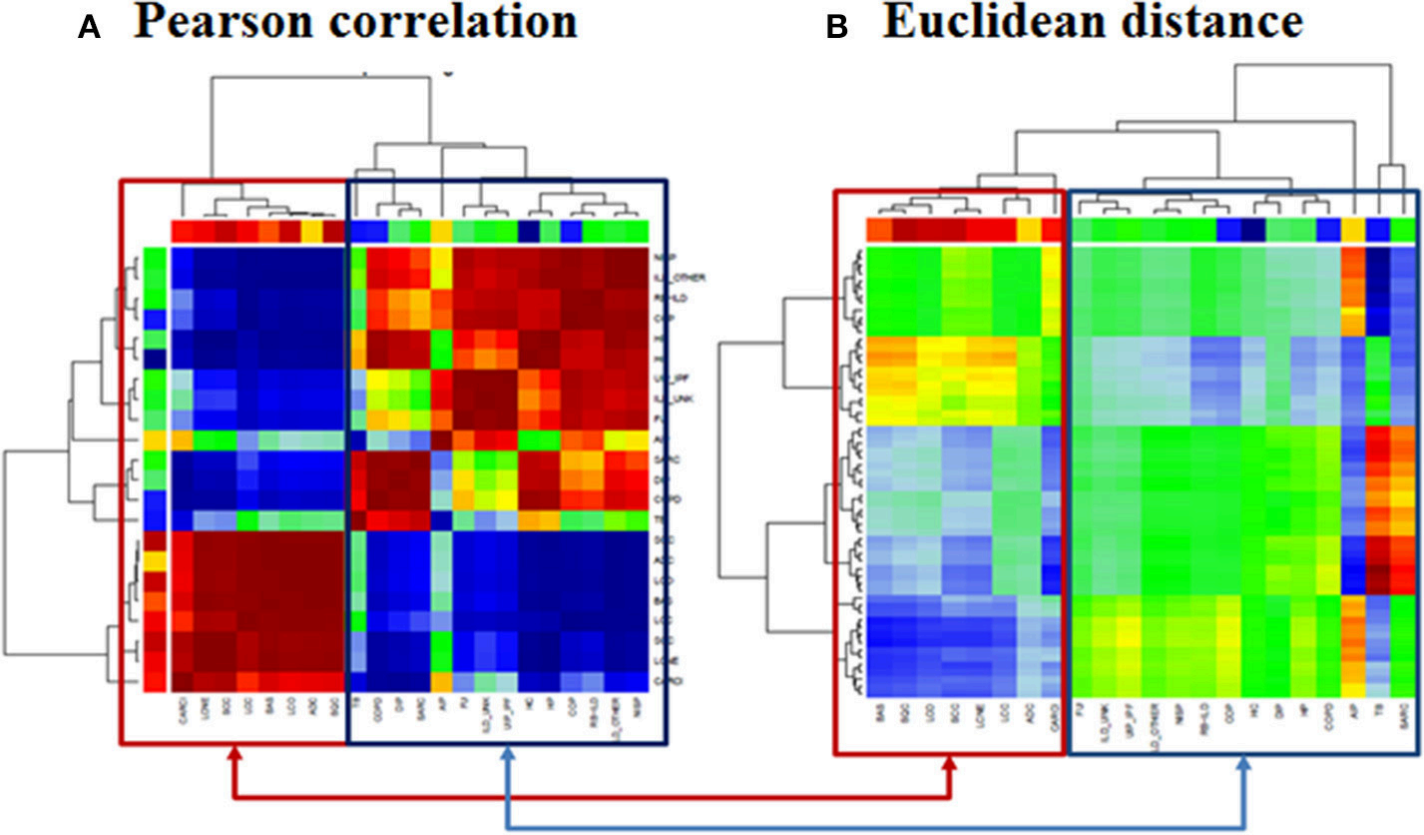
**Clustering of lung diseases and healthy controls**. Clustering was performed with **(A)** Pearson correlation and **(B)** Euclidean distance metrics. In both cases we observed two main clusters collecting mainly cancer and non-cancer samples, respectively (see the color bars which assign the samples to the diseases according to color code legend in Figure 2).

**Figure 7:**
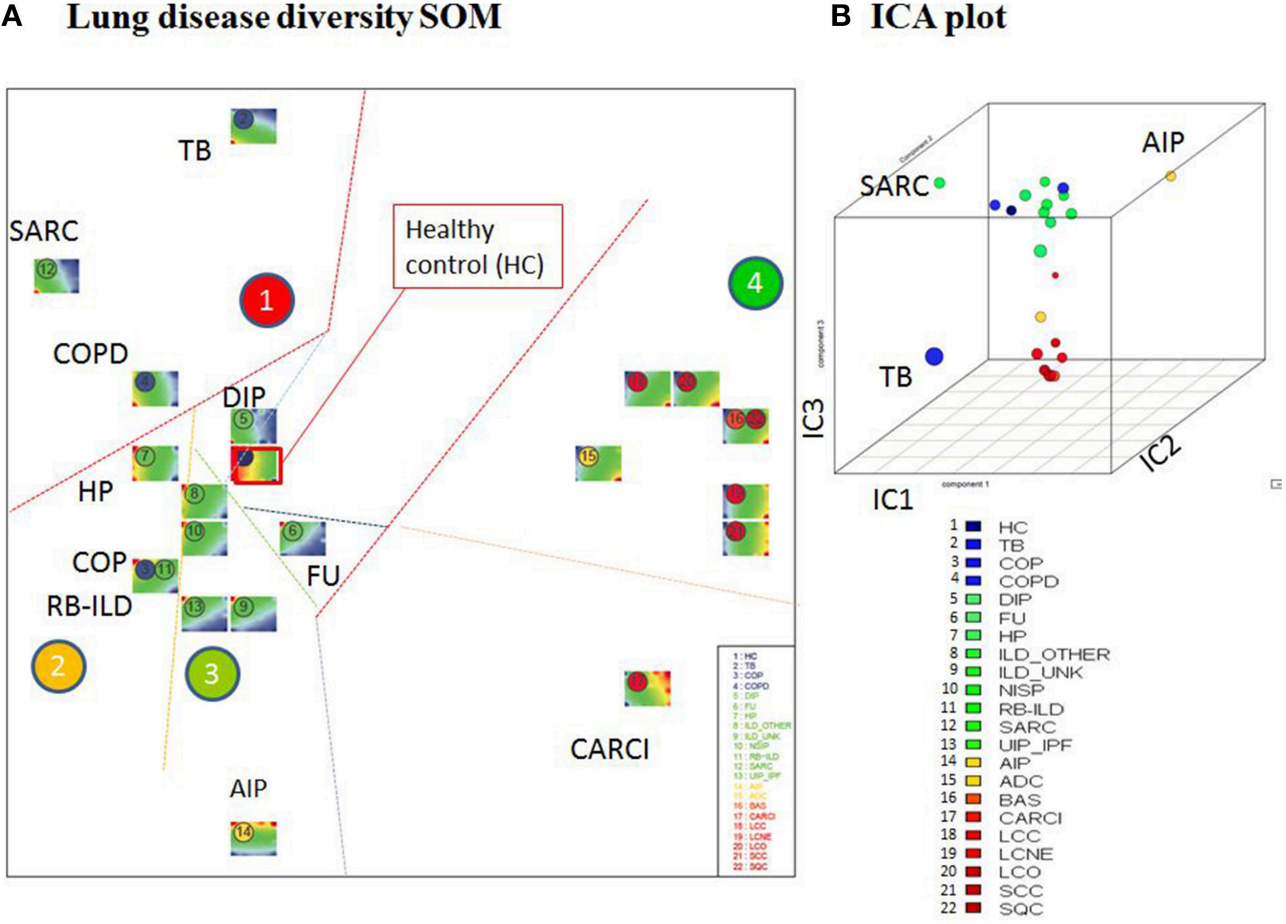
**Diversity analysis of lung diseases. (A)** The sample-diversity SOM projects the multidimensional diversity landscape into a two-dimensional plot. Each disease class is visualized by its sample portraits. Borders between the clusters 1–4 serve as guides for the visual inspection. **(B)** The three dimensional ICA plot shows that independent component 3 (IC3) mainly separates lung cancers from chronic lung diseases.

Visual inspection of the SOM portraits revealed considerable similarities among non-cancer, as well as cancer diseases. Hierarchical cluster analysis of the SOM portraits using either Pearson correlation or Euclidian distances of meta-PSF values as similarity metrics revealed two main clusters of diseases. In both cases, the first cluster grouped lung cancer diseases, while the second cluster contained non-cancer diseases and healthy controls (Figures 6A,B). For a higher resolution of the diversity analysis we applied SOM mapping of the samples (so-called 2nd level SOM) and ICA as described in Wirth et al. (2012a). The SOM mapping reveals that especially the cluster of non-malignant lung diseases further disentangles into different subclusters labeled 1–3 (Figures 7A,B). Similarly, the 2nd level SOM, ICA, which assumes non-Gaussian distribution of distances between samples, clearly separates cancer from non-cancer lung diseases. TB, SARC, and AIP appear as outliers that are separated from the other diseases.

### Pathway deregulation profiles in lung diseases compared to healthy controls

**Figure 8:**
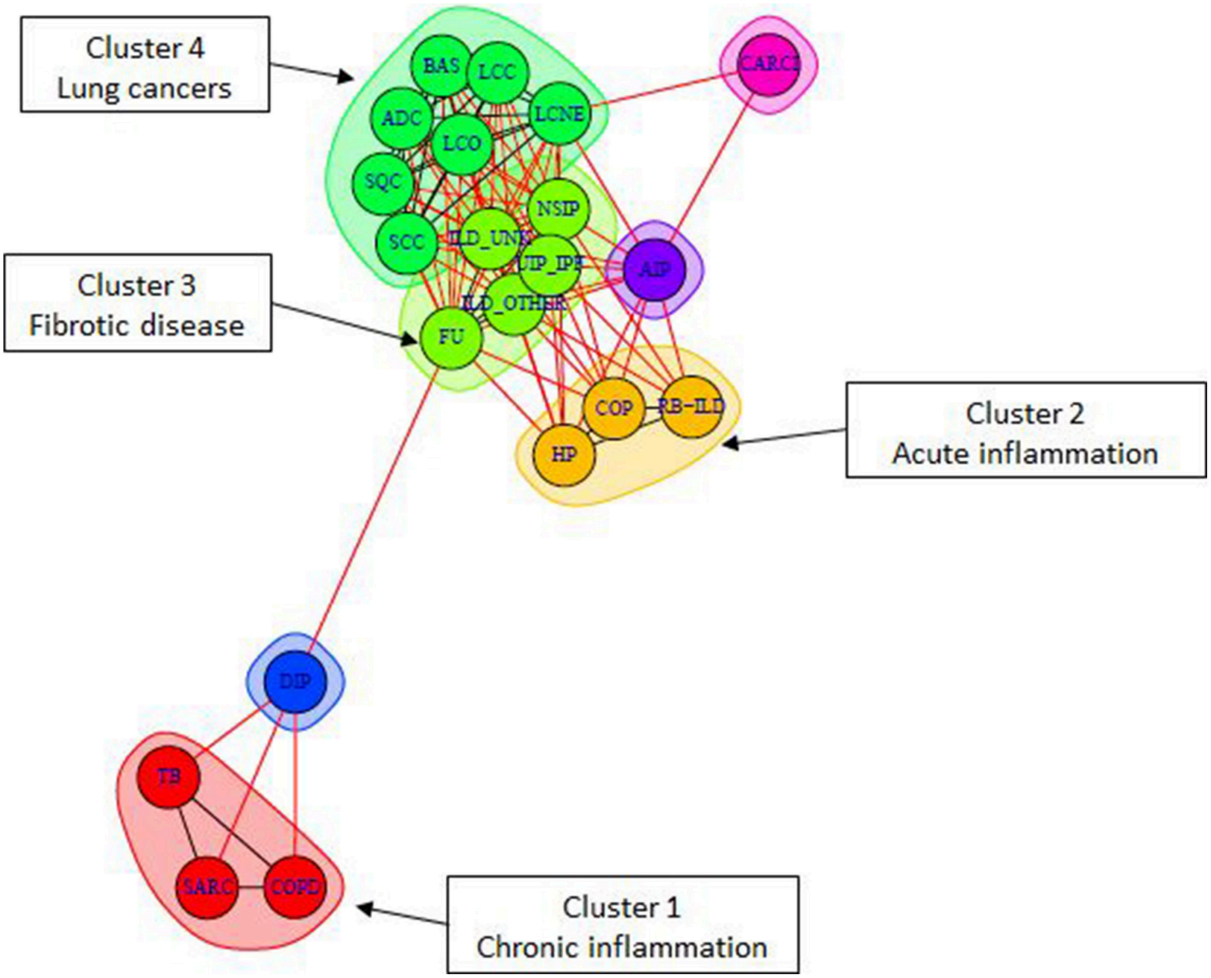
**Community detection in the lung disease graph**. Each color represents one community, which gathers diseases with the highest pairwise similarity in pathway deregulation states (see also Table 2). The graph layout is generated with Fruchterman–Reingold algorithm where edge length is inversely correlated with node connectivity.

In order to assess pathway activity deregulations in lung diseases compared to healthy state, we calculated differential PSF values respective to healthy controls. Most of the obtained differential SOM portraits showed similar spot distributions compared to the original ones (see Supplementary Material Data Sheet 12, Supplementary Figure 1). Next, we created a graph object with nodes representing diseases and edges associated with the number of co-regulated pathways between each pair of disease nodes. Finally, we performed a graph community search with random walktrap algorithm in order to identify diseases sharing maximal similarities in pathway deregulation states. This algorithm analyzes the graph by random walks to identify densely connected subgraphs, called communities. In total, we identified 4 communities (or clusters) containing three or more diseases (Figure 8 and Table 2, clusters 1–4). Each multi-disease community is specifically described by shared pathway sinks, similarly deregulated in all members of a given community (Table 2). Particularly, we observed that diseases in the cancer cluster contained 14 shared pathway deregulation states, while chronic lung disease clusters showed less number of shared states (between three and five).

**Table 2:** **Assessment of pathway common deregulation profiles in lung disease communities**.

Community (color^*^)	**Diseases**	**Deregulated pathway**	**Sink (GeneID/Symbol)**	**Associated molecular process**
Cluster 1 (Red)	SARC TB COPD	B cell receptor signaling pathway	FOS/2353	Immune response (UP)
		Chemokine signaling pathway	WAS/7454	Regulation of cytoskeleton (UP)
		NF-kappa B signaling pathway	CCL19/6363	Lymphoid-tissue homing (UP)
		Toll-like receptor signaling pathway	CCL3L3/414062	Chemotactic effect (UP)
Cluster 2 (Orange)	RB-ILD HP COP	Hippo signaling pathway	SERPINE1/5054	Antiapoptosis (DN)
Cluster 3 (Light green)	UIP_IPF ILD_OTHER ILD_UNK NSIP FU	Cytokine-cytokine receptor interaction	TNFRSF17/608	TNF Family (UP)
		Cytokine-cytokine receptor interaction	CXCR5/643	CXC Family (UP)
		Wnt signaling pathway	MMP7/4316	Cell cycle (UP)
Cluster 4 (Green)	ADC SQC LCC LCO BAS LCNE SCC	Alanine, aspartate, and glutamate metabolism	GFPT1/2673	Amino sugar metabolism (UP)
		Alanine, aspartate, and glutamate metabolism	ASPA/443	L-aspartate (UP)
		Glycine, serine, and threonine metabolism	AOC2/314	Methylglyoxal (DN)
		Glycolysis/Gluconeogenesis	PGK1/5230	Glycerate1,3P (UP)
		Purine metabolism	PGM1/5236	Ribose-5P (UP)
		Purine metabolism	GDA/9615	Guanine (UP)
		Pyrimidine metabolism	TK1/7083	dUMP(UP)
		Adrenergic signaling in cardiomyocytes	AKT3/10000	Apoptosis (DN)
		p53 signaling pathway	IGF1/3479	Apoptosis (DN)
		p53 signaling pathway	GTSE1/51512	DNA damage prevention & repair (UP)
		Progesterone-mediated oocyte maturation	ANAPC10/10393	Metaphase arrest (DN)
		Progesterone-mediated oocyte maturation	CDK1/983	Cell cycle (UP)

* *DIP, CARCI, and AIP formed separate single member communities; UP, up-regulation; DN, downregulation*.

The first community (Cluster 1—red) of non-cancer lung diseases contained COPD, sarcoidosis (SARC), and tubercolosis (TB). Numerous experimental studies, including our own, implicated immune/inflammatory pathways such as Toll-like receptor signaling, phagocytosis, and chemokine signaling in sarcoidosis, tuberculosis, and COPD (Arakelyan et al., 2009; Haspel and Choi, 2011; Kriegova et al., 2011; An et al., 2012; Maertzdorf et al., 2012; Pabst et al., 2013; Pugazhendhi et al., 2013). Moreover, differential gene expression as well as gene polymorphisms associated with these diseases demonstrated considerable mutual overlap (Arakelyan et al., 2009; Haspel and Choi, 2011). Furthermore, the pathophysiology of TB, SARC, and COPD is very similar, often making them hard to distinguish (Maertzdorf et al., 2012). Thus, our findings are in line with previous findings confirming the presence of shared deregulation of immune system pathways in these diseases.

The second community (Cluster 2—orange) joined respiratory bronchiolitis interstitial lung disease (RB-ILD) (Leslie, 2009; Meyer et al., 2012), hypersensitivity pneumonia (HP), and cryptogenic organizing pneumonia (COP). The molecular pathways involved in the development of these diseases are poorly understood and there is not much data on gene expression changes in these disorders. Nevertheless, the clustering of RB-ILD, HP, and COP together based on global gene expression has also been described previously (Cho et al., 2011; Lee and Yang, 2013). Our results showed that, these diseases were characterized by shared downregulation of anti-apoptotic and proinflammatory branches of Hippo signaling pathway. This pathway is an important regulator of tissue growth. It has been studied largely in tumor development, while results on non-cancer lung diseases are mostly limited to animal models (Gomez et al., 2014). It has been shown that the activation of Hippo signaling pathway in non-cancer diseases is beneficial because it promotes tissue regeneration through proliferation and inhibition of apoptosis (Halder and Johnson, 2011). By contrast, inhibition of Hippo signaling can promote substitution of healthy tissues with fibrotic tissue during cardiac remodeling (Xin et al., 2013). Thus, we can speculate that this pathway may be implicated in progression of fibrosis in these diseases.

The third and the biggest community of interstitial lung diseases (Cluster 3—light green) included interstitial pneumonias, such as UIP/IFP, NSIP, and unclassified interstitial lung diseases (ILDs). This cluster was characterized by activation of various cytokine-cytokine receptor interactions, and upregulation of WNT signaling pathway. Moreover, it has been noticed that this cluster serves as a link between cancer and other ILD diseases (Archontogeorgis et al., 2012; Vancheri, 2013). The observed association between ILD, and especially UIP/IPF with cancer, has been reported previously (reviewed in Archontogeorgis et al., 2012; Vancheri, 2013). It has been noted that IPF and lung malignancies share molecular pathomechanisms, including epigenetic changes, delayed apoptosis and changes in cell-cell interactions (Vancheri, 2013). Our results demonstrated that the link can be realized through common upregulation in cell cycle related pathways (Hippo, p53), signal transduction (cytokine, thyroid hormone), and metabolism of nucleic acids (purines and pyrimidines).

Finally, cancer diseases (Cluster 4—green) were clustered into one highly homogeneous community characterized by downregulation of immune system related pathways and upregulation of cell cycle, proliferation, and metabolism pathways, as expected (Han et al., 2014; Domagala-Kulawik, 2015). From all lung cancer types included in our dataset, the lung carcinoid tumor (CARCI) demonstrated a different pathway deregulation profile (Anbazhagan et al., 1999), which may indicate its neuroendocrine origin (Rekhtman, 2010). Indeed, our data shows that it shares considerable amount of similarities with large cell neuroendocrine cancer (Figure 8).

Besides the multi-disease communities, we observed the presence of “linker” disease nodes (DIP, CARCI, and AIP) in the similarity graph. Though they are not included in any cluster, they provide links between different clusters.

The community detection analysis confirmed the diverse nature of cancer and non-malignant lung diseases, but it also provided additional information that wasn't obvious using other similarity analysis methods, such as clustering, second level SOM or ICA. The main advantage here was that the graph object allowed for direct assessment of similarly deregulated pathways between all pairs of studied diseases, with reference to the healthy state. We have noticed that pathway deregulation states in fibrotic diseases (cluster 3) serve as a central hub that shares considerable similarities with inflammatory (cluster 1, 2) and cancer diseases (cluster 4). Moreover, our results suggest that interstitial lung diseases seem to constitute a more heterogeneous group of diseases in terms of molecular mechanisms underlying their pathology. Even if it is well known that inflammation and fibrosis are the main characteristics of interstitial lung diseases (Bourke, 2006), our data suggested that the drivers of these processes are different and are associated with perturbations in different pathways.

## Discussion

Using our novel PSF-SOM method we have evaluated similarities of pathway deregulation profiles in a large spectrum of lung pathologies by mining high-dimensional gene expression data and topologies of signaling and metabolic pathways. We pursued a systems biology view to identify most prominent properties of groups of lung diseases in terms of pathway deregulation patterns. Our results revealed considerable differences in pathway deregulations implicated in cancer and non-cancer lung diseases. While lung cancers were characterized by pathways implicated in cell proliferation, metabolism, non-malignant lung diseases were characterized by deregulations in pathways involved in immune/inflammatory response and fibrotic tissue remodeling. Moreover, we observed considerable heterogeneity in terms of pathway deregulation in interstitial lung diseases (ILD). We were able to identify three groups of ILDs and showed that the development of characteristic pathological processes such as fibrosis can be initiated by deregulations in different signaling pathways. These results raise a question whether it is appropriate to combine ILDs into single group of diseases. Finally, we also detected substantial similarities between cancers and a subset of interstitial lung diseases characterized by fibrosis development, which suggests the presence of shared molecular mechanisms involved in their pathogenesis.

Our results replicate existing knowledge on pathological processes in lungs. The inflammation and fibrosis related pathways identified to be deregulated in non-malignant chronic lung diseases considerably overlapped with the ones reported by Cho et al. (2011) using gene-centered analysis approaches (see Additional File 1 of Cho et al., 2011). Moreover, deregulations of metabolic activities and cell cycle control have also been reported previously in lung cancers (Cantor and Sabatini, 2012). Furthermore, our findings are in line with the results of recent publication by Kim et al. (Cantor and Sabatini, 2012) on interstitial disease subgrouping based in molecular phenotypes. However, we believe that our study has a number of important differences compared to the previously reported results. In their paper, authors used ANOVA significance value cut-offs for identification of differentially expressed genes and have applied topology free enrichment analysis for functional assessment of biological processes implicated in lung diseases. Consequently, all the criticism we have mentioned in the Introduction, also applies here. In contrast, our results directly extend current understanding of molecular events associated with various aspects of lung pathology. We did not limit our study with obstructive or interstitial lung diseases only, but aggregated lung cancers, chronic non-cancer lung diseases and, especially, a wide spectrum of interstitial lung diseases. Moreover, we have directly evaluated pathway activity deregulation states for each ILD type separately, rather than obtaining results about interstitial diseases in general. This is a special feature of this work and our data may provide additional insight into pathobiology of these frequently neglected diseases, which are often difficult to diagnose and treat.

From methodological point of view this paper demonstrates the power of our pathway centered analysis. While usual gene-centered studies provide lists of genes and associated functional categories, our approach more closely refers to systems level definitions and provides a rich outlook on actual molecular events associated with studied conditions. We have combined PSF and SOM algorithms that previously have been extensively benchmarked (Wirth et al., 2011, 2012a; Binder et al., 2014; Löffler-Wirth et al., 2015; Nersisyan et al., 2015). The PSF algorithm integrates pathway knowledge and topology information with gene expression data to identify deregulated branches of pathways. On the other hand, SOM analysis allows for mining for pathways with similar deregulation patterns across datasets.

This study has a number of limitations that is worth to discuss here. First, the data analyzed in this study refer to endpoint pathologies, and not to initial steps of disease development. While the latter is important for assessing the causes of a disease, the former describes the state at which most therapeutic interventions are targeted. Thus, assessment of disease similarities based on end-point phenotypes is not suited to study initiation and early genesis of lung diseases. Second, our study was limited to KEGG pathways that contain previously curated information (Ogata et al., 1999). This approach does not allow for identification of new gene interactions or pathways implicated in lung diseases. On the other hand, it provides the advantage of using “gold standard” pathway topologies, which are enriched with information about known functional outcomes.

In conclusion, this paper describes the pathobiology of lung diseases from systems viewpoint using a pathway centered high-dimensional data mining approach. Our results largely contribute to current understanding of pathological events in lung cancers and non-malignant lung diseases. Moreover, our results provide new insight into molecular mechanisms of a number of interstitial lung diseases that have been studied to a lesser extent compared to interstitial pulmonary fibrosis, COPD, and sarcoidosis.

## Author contributions

AA, LN, and HB initiated the study. AA and LN performed calculations with contribution from HB and HL. AA, LN, HB, HL, and MP contributed to results interpretation and manuscript writing. All authors read edited and approved the final manuscript.

### Conflict of interest statement

The authors declare that the research was conducted in the absence of any commercial or financial relationships that could be construed as a potential conflict of interest.

## Abbreviations

ANOVA: analysis of variance
FC: Fold Change
FDR: False Discovery Rate
GEO: Gene Expression Omnibus
GO: Gene Ontology
ICA: Independent Component Analysis
ILD: Interstitial Lung Disease
KEGG: Kyoto Encyclopedia of Genes and Genomes
PSF: Pathway Signal Flow
SOM: Self-Organizing Maps.
