# Supplementary material for: Cartography of Pathway Signal Perturbations Identifies Distinct Molecular Pathomechanisms in Malignant and Chronic Lung Diseases
Source: Front Genet. 2016 May 6;7:79. doi: 10.3389/fgene.2016.00079 (PMC4859092; doi:10.3389/fgene.2016.00079)
Supplement: Supplementary file 2 [file DataSheet2.doc]

Data Sheet 2

# Cartography of pathway signal perturbations identifies distinct molecular pathomechanisms in malignant and chronic lung diseases

Arsen Arakelyan*, Lilit Nersisyan, Martin Petrek, Henry Löffler-Wirth, and Hans Binder

*** Correspondence:** Arsen Arakelyan: aarakaleyan@sci.am

**Data, Scripts and Data Analysis Raw Results**

All datasets, scripts and analysis raw results can be found under the following link:

<https://www.dropbox.com/sh/l5ndkef99tf8dki/AABypF91hmCc-991pO6e63lXa?dl=0>

**Contents**

Data Folder: **psf_data**

| File/Folder name | Description |
| --- | --- |
| GSE_preproc | FC over the mean expression value across all the control (healthy) samples included in the same dataset |
| Phenotypes | Sample descriptions with names, abbreviations and other details |
| pathway.names.txt | Indicator whether a pathway can be used in PSF analyses (0 - not used, 1 - used) |
| pathList.Rdata | KEGG Pathway IDs |
| KEGG.Collection.Rdata | A collection of graph objects representing KEGG pathway maps used as the pathway topology source for PSF analysis |
| Indata.Rdata | The final matrix containing log10 tranfromed PSF values supplied to oposSOM as an input |
| comms_colors.txt | Assignment of diseases to graph walktrap communities (clusters) |

Scripts Folder: **psf_scripts**

*If you intend to repeat our entire calculations, set the* working directory here!!!

| Name | Description |
| --- | --- |
| **For PSF calculation** | |
| import.packages.R | Installs all required r packages from CRAN or Bioconductor repositories  *Required packages and repositories:*  *oposSOM bioconductor*  *IRanges bioconductor*  *Rgraphviz bioconductor*  *graph bioconductor*  *graphite bioconductor*  *biomaRt bioconductor*  *GEOquery bioconductor*  *igraph cran*  *fdrtool cran* |
| psf_lung.R | The main script for PSF calculation |
| geneidconversions.R | Converts gene official symbols to Entrez IDs and back |
| pathnets.R | Prepares data for calculation |
| pathnets.functions.R | Functions for PSF calculation |
| psf.R | Functions for PSF calculation |
| **For post SOM data analyses** | |
| exrtact.filtered.metadata.R | Extracts spot-pathway information |
| sig.pairwise.R | Comparison for PSF between lung diseases and healthy lungs |
| sd.estimatioin.R | Significance estimation for pairwise comparisons |
| pairvise.graph.plot.R | Creation of a disease graph, and identification of disease communities (clusters) |
| extract.common.pathways.R | Identification of common pathway activity deregulations within graph communities (clusters) |

Data Folder: **oposSOM_data**

| File/Folder name | Description |
| --- | --- |
| lung.psf.log.replicatesQTNF_C5_80_S90_final - Results | Final results for SOM analysis of the PSF processed dataset |
| lung.psf.log.replicatesQTNF_C5_80_S90_final.RData | The SOM environment R data |
