# Supplementary figures and images for: Cartography of Pathway Signal Perturbations Identifies Distinct Molecular Pathomechanisms in Malignant and Chronic Lung Diseases
Source: Front Genet. 2016 May 6;7:79. doi: 10.3389/fgene.2016.00079 (PMC4859092; doi:10.3389/fgene.2016.00079)

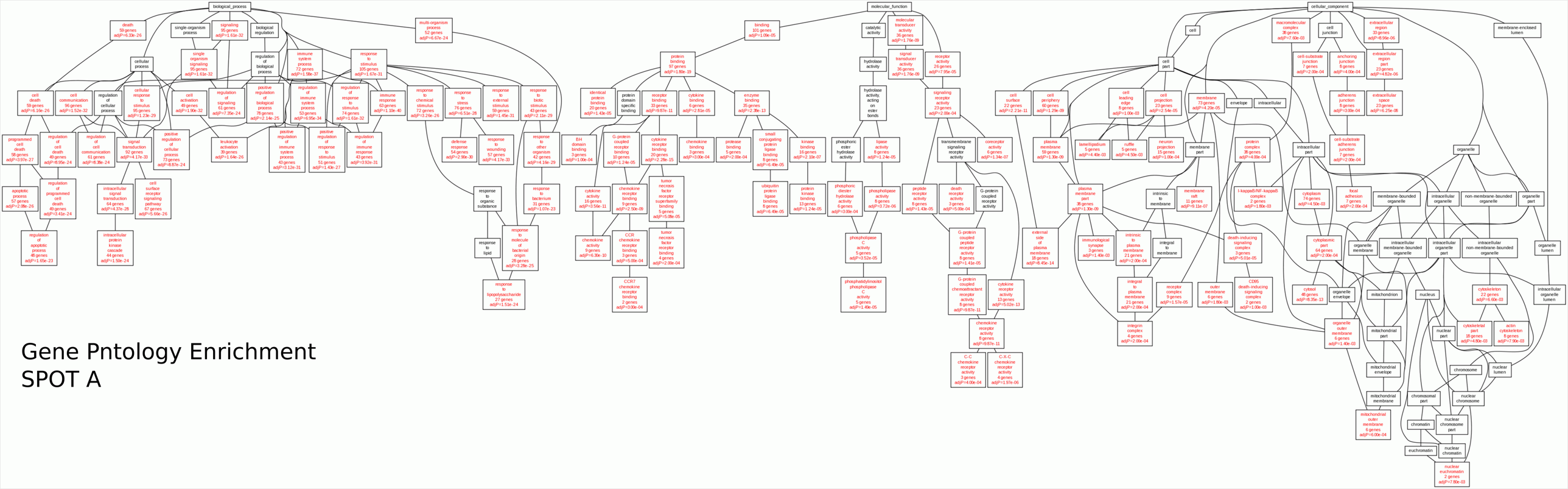

# Gene Pntology Enrichment SPOT A

Supplement: Supplementary file 8 [file DataSheet8.pdf]

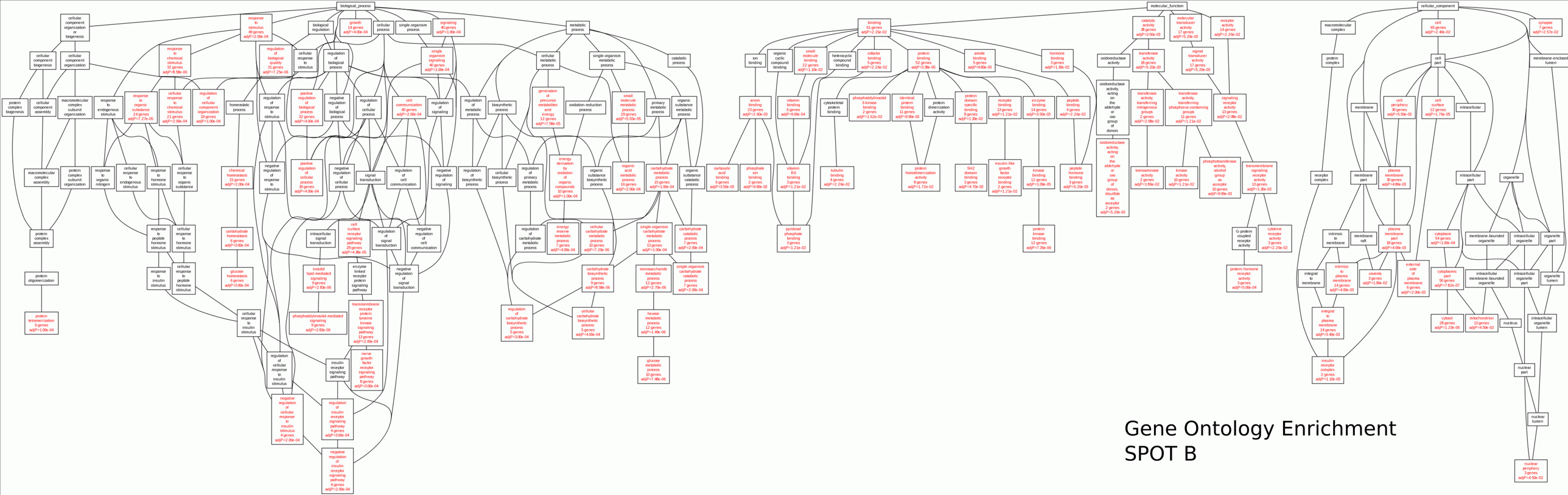

Supplement: Supplementary file 9 [file DataSheet9.pdf]

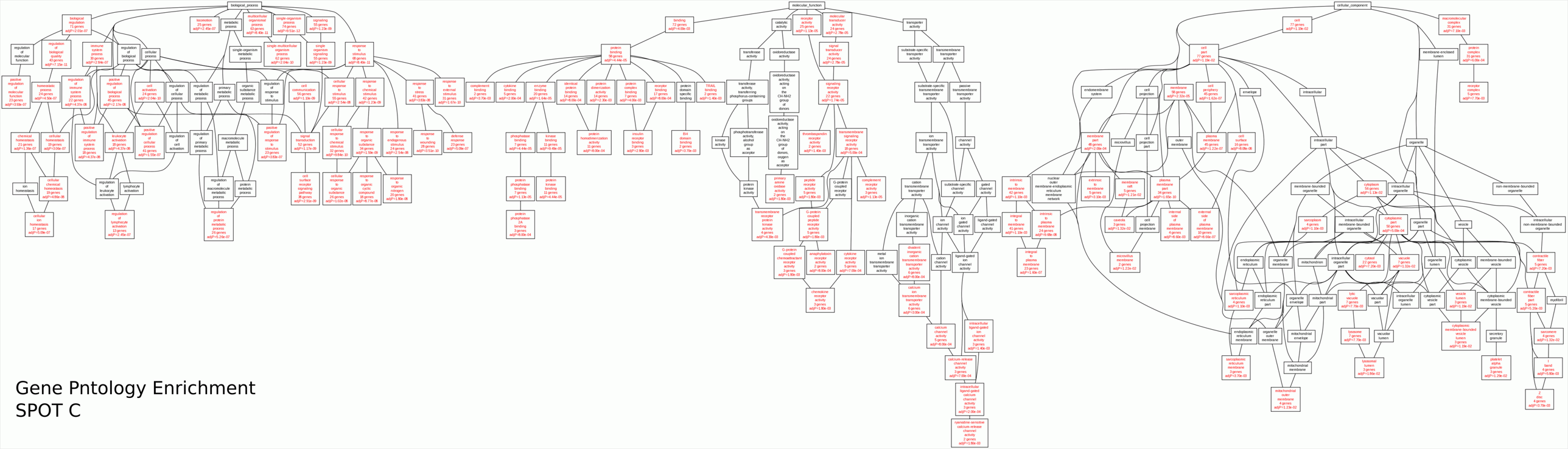

Gene Ontology Enrichment  
SPOT C

Supplement: Supplementary file 10 [file DataSheet10.pdf]

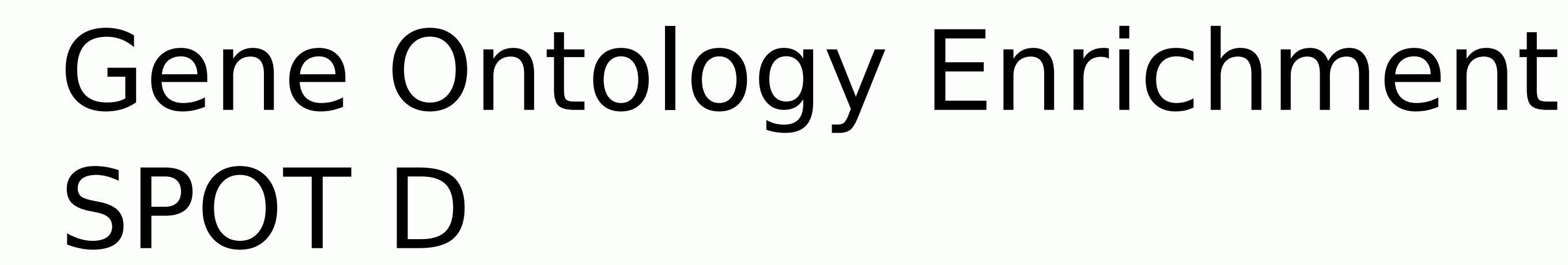

Supplement: Supplementary file 11 [file DataSheet11.pdf]
