# Supplementary material for: Cartography of Pathway Signal Perturbations Identifies Distinct Molecular Pathomechanisms in Malignant and Chronic Lung Diseases
Source: Front Genet. 2016 May 6;7:79. doi: 10.3389/fgene.2016.00079 (PMC4859092; doi:10.3389/fgene.2016.00079)
Supplement: Supplementary file 12 [file DataSheet12.docx]

***Data Sheet 12***

**Cartography of pathway signal perturbations identifies distinct molecular pathomechanisms in malignant and chronic lung diseases**

**Arsen Arakelyan*, Lilit Nersisyan, Martin Petrek, Henry Löffler-Wirth, and Hans Binder**

*** Correspondence:** Arsen Arakelyan: aarakaleyan@sci.am


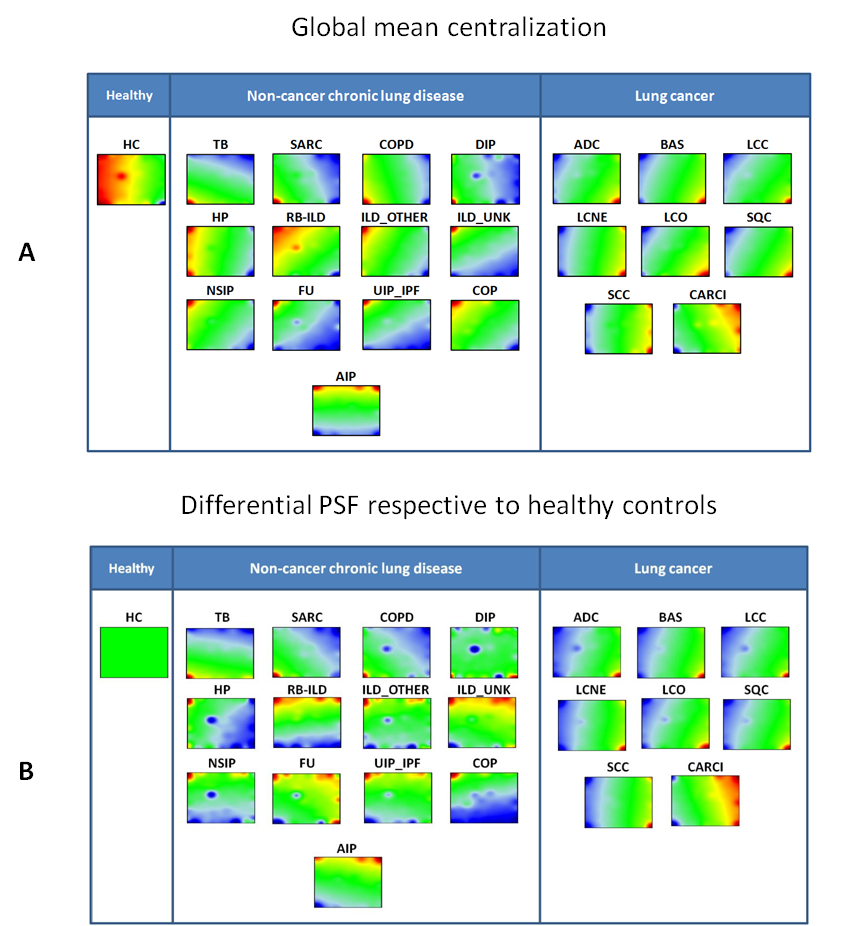


**Supplementary figure 1.** Spot distributions in lung disease SOM "portraits" respective to pathway sink global mean normalization **(A)** and respective to healthy lung state **(B)**. For details, see Methods section of the manuscript.
